# Apoptosis-associated speck-like protein containing a CARD-mediated release of matrix metalloproteinase 10 stimulates a change in microglia phenotype

**DOI:** 10.3389/fnmol.2022.976108

**Published:** 2022-10-11

**Authors:** Kathryn E. Sánchez, Kiran Bhaskar, Gary A. Rosenberg

**Affiliations:** ^1^Center for Memory and Aging, University of New Mexico, Albuquerque, NM, United States; ^2^Department of Molecular Genetics and Microbiology, University of New Mexico, Albuquerque, NM, United States; ^3^Department of Neurology, University of New Mexico, Albuquerque, NM, United States

**Keywords:** inflammasome, apoptosis-associated speck like protein containing a CARD (ASC), matrix-metalloproteinase 10 (MMP10), microglia, cytokine

## Abstract

Inflammation contributes to amyloid-β and tau pathology in Alzheimer’s disease (AD). Microglia facilitate an altered immune response that includes microgliosis, upregulation of inflammasome proteins, and elevation of matrix-metalloproteinases (MMPs). Studies of cerebrospinal fluid (CSF) and blood in dementia patients show upregulation of two potential biomarkers of inflammation at the cellular level, MMP10 and apoptosis-associated speck-like protein containing a CARD (ASC). However, little is known about their relationship in the context of brain inflammation. Therefore, we stimulated microglia cultures with purified insoluble ASC speck aggregates and MMP10 to elucidate their role. We found that ASC specks altered microglia shape and stimulated the release of MMP3 and MMP10. Furthermore, MMP10 stimulated microglia released additional MMP10 along with the inflammatory cytokines, tumor-necrosis factor-α (TNFα), Interleukin 6 (IL-6), and CXCL1 CXC motif chemokine ligand 1 (CXCL1). A broad-spectrum MMP inhibitor, GM6001, prevented TNFα release. With these results, we conclude that MMP10 and ASC specks act on microglial cells to propagate inflammation.

## Introduction

Fifty-five million people in the world are currently affected by dementia, and this number is predicted to double every 20 years according to the World Health Organization. It will eventually reach 78 million by 2030 and 139 million by 2050 due to a rapidly aging population (Prince et al., 2013). The expected rise in dementia is concerning since patient quality of life is greatly diminished by symptoms, which include agitation, apathy, aggression, psychosis, hallucinations, and delusions. Though the presence of amyloid-β and tau neurofibrillary tangles in the brain are canonical pathological hallmarks of Alzheimer’s disease, these two pathological proteins are often associated with central nervous system inflammation. More specifically, patient samples demonstrate evidence of altered microglial activity and elevated cytokine levels. Such microglial activity includes microgliosis, the upregulation of inflammasome adaptor proteins, and elevated MMP levels (Ueno et al., 2009; Kerkhofs et al., 2020; Scott et al., 2020; Erhardt et al., 2021; Jiang et al., 2021).

The NLRP3 inflammasome is a multiprotein complex that is comprised of the following proteins: NACHT, LRR, and PYD domain-containing protein 3 (NLRP3); the adaptor protein apoptosis-associated speck-like protein containing a CARD (ASC); and inflammatory caspase 1 (cysteine-dependent aspartate-directed protease 1) (Latz et al., 2013; Rathinam and Fitzgerald, 2016; Reis et al., 2020; Zheng et al., 2020). The ASC and pro-caspase 1 components of the complex lead to caspase 1 activation that results in the processing of cytoplasmic targets, including IL-1β and IL-18 (Sutterwala et al., 2006; Franklin et al., 2014; Facci et al., 2018; Ribeiro et al., 2020; Scott et al., 2020). The NLRP3 inflammasome complex is predicted to contribute to AD, as it promotes neuroinflammation in response to amyloid-β. Assembled ASC (called ASC speck or pyroptosome) has prion-like activity, and is capable of propagating inflammation between immune cells (Franklin et al., 2014). One important inflammasome adaptor protein, ASC, is elevated in the immune cells of AD patients and is associated with the inflammasome complex (Franklin et al., 2014; Scott et al., 2020). ASC specks recruit and activate caspase-1, which results in 1β (IL-1β) cleavage and pyroptotic cell death (Franklin et al., 2014; White et al., 2017; Scott et al., 2020). Immune cells then release ASC specks into the extracellular space, which can further propagate inflammation by maturing IL-1β and IL-18 (Venegas et al., 2017). Finally, our previous research showed elevated ASC specks in the CSF of patients with tauopathies compared to healthy subjects (Jiang et al., 2021).

While it is still unclear how inflammasome-based maturation of IL-1β is related to another important mediator of inflammation, matrix metalloproteinase (MMPs), previous research suggests that IL-1β stimulates the release of MMP1, MMP9, and MMP13 in chondrocytes (Zhang et al., 2018). Furthermore, MMP1 is also upregulated in fibroblasts in response to IL-1β (Lew et al., 2018). These studies emphasize a likely relationship between inflammasome activation and MMP release. However, how the prion-like ASC speck, an inflammatory protein that is a prerequisite for IL-1β activation, can affect MMP expression is still unknown.

Matrix-metalloproteinases are endopeptidases that are elevated in Alzheimer’s disease (Lorenzl et al., 2003; Rosenberg, 2009; Erhardt et al., 2021). For example, MMP9 and MMP10 are elevated in the CSF of patients with dementia (Lorenzl et al., 2003; Erhardt et al., 2021; Jiang et al., 2021). The elevation of MMPs is notable since they cleave numerous targets including extracellular matrix proteins, G-protein coupled receptors, and tight junction proteins (Yang et al., 2007; Ueno et al., 2009; Yang and Rosenberg, 2011; Allen et al., 2016). The innate immune cells of the central nervous system, microglia, respond to fluctuations in MMP activity. For example, MMP13 and MMP3 both alter the inflammatory profile of microglia (Connolly et al., 2016; Sánchez and Maguire-Zeiss, 2020; Brelstaff et al., 2021). MMP13 exposure changes microglia morphology and proinflammatory protein expression (Sánchez and Maguire-Zeiss, 2020). MMP3 is a stromelysin like MMP10, and can stimulate the upregulation and release of the proinflammatory cytokine TNFα in microglia cell lines (Kim et al., 2005; Connolly et al., 2016). While MMP3 is investigated in this work, it is notably not as novel as MMP10 due to these previous studies.

Though it is well established that both inflammasome adaptor proteins and MMPs contribute to the inflammation documented in Alzheimer’s disease, the relationship between assembled ASC specks and MMP10 has not been investigated in microglia. To that end, we have evaluated the consequences of ASC speck stimulation on primary microglia. After confirming that MMP10 is released in response to ASC specks, we also assessed how MMP10 further perpetuates microglia-mediated inflammation. Here, we report that MMP10 stimulates a pro-inflammatory response in microglia. Overall, our findings have identified the inflammasome as a future therapeutic target to combat MMP mediated inflammation.

## Materials and methods

### Animals

The use of C57Bl/6j pups for primary microglia culture and all other animal work was performed at the University of New Mexico Health Sciences Center where the protocol for such experiments were approved by the Institutional Animal Care and Use Committee (IACUC).

### Culture of ASC-mCerulean macrophages

Inflammasome reporter macrophages stably transduced with constructs for the expression of ASC-mCerulean have been previously described (Heneka et al., 2013; Stutz et al., 2013; Franklin et al., 2014; Jiang et al., 2021). The immortalized macrophages were grown in DMEM (Thermo Fisher) supplemented with 10% v/v Fetal Bovine Serum (FBS; Thermo Fisher Fisher). When cells reached 60% confluence (P8-P12), they were seeded on petri dishes (100 mm; Thermo Fisher Scientific) in preparation for ASC-speck purification.

### Purification of ASC specks

ASC-mCerulean expressing macrophages were grown in a 10-mm petri dish and stimulated with lipopolysaccharide (LPS) (250 ng/mL; InvivoGen) for 3 h. Without removing the cell-culture media, macrophages were then activated 1 h with nigericin (20 μM; InvivoGen) as previously described (Fernandes-Alnemri and Alnemri, 2008; Stutz et al., 2013; Franklin et al., 2014). Macrophages were subsequently harvested and lysed in 0.5 mL lysis buffer (20 mM Hepes-KOH, pH 7.5, 10 mM KCl, 1.5 mM MgCl_2_, 1 mM EDTA, 1 mM EGTA, 320 mM sucrose) and kept on ice for 30 min. The lysate was subsequently centrifuged for 10 min to remove the bulk nuclei (1500 rpm; Eppendorf Centrifuge 5415 R). The resulting supernatant was transferred to a new tube and diluted with 0.5 mL lysis buffer and 1 mL CHAPS buffer (20 mM Hepes-KOH, pH 7.5, 5 mM MgCl_2_, 0.5 mM EGTA, 0.1 mM PMSF, 0.1% CHAPS). After dilution, samples were centrifuged for 10 min to pellet the ASC pyroptosomes (5,000 rpm). For further purification, the crude pellet was resuspended in 1 mL CHAPS buffer and subsequently transferred to a new tube when layered over a 40% Percoll cushion (1 mL; 40% v/v Percoll in 2.5 M sucrose) and centrifuged at 14,000 rpm for 10 min as previously established (Stutz et al., 2013). Lastly, the ASC-specks were resuspended in CHAPS buffer for further use in cell culture experiments.

### Primary microglia culture

A minimum of five C57BL/6j pups were used for all dissections and experiments, and postnatal (P0-3) mouse pup cortices were used to generate primary microglia cultures (Daniele et al., 2014, 2015; Sánchez and Maguire-Zeiss, 2020). Cortices were dissected, subsequently homogenized, and grown in a T75 flask for 16-21 days in Microglia Culture Media (MCM; 1× Minimal Essential Medium Earle’s (MEM) supplemented with: 1 mM L-glutamine, 1 mM sodium pyruvate, 0.6% v/v D-(+)-glucose, 100 μg/ml Penicillin/Streptomycin (P/S), 4% v/v Fetal Bovine Serum (FBS), 6% v/v Horse Serum). To enrich for microglia, flasks were shaken for 3 h, and the cells of all pups were mixed immediately before plating. Cells were then plated in microglia growth media (MGM) containing 5% v/v Fetal Bovine Serum (MGM; Minimum Essential Medium Earle’s (MEM), supplemented with 1 mM sodium pyruvate, 0.6% (v/v) D-(+)-glucose, 1 mM L-glutamine, 100 μg/mL penicillin/streptomycin, and 5% v/v Fetal Bovine Serum) (Béraud et al., 2011, 2013; Daniele et al., 2014, 2015; Sánchez and Maguire-Zeiss, 2020). For all experiments, microglia were plated in a 24-well format at a minimum density of 5.0 × 10^4^ on sterile glass coverslips (12 mm; Deckglaser).

### Stimulation of primary microglia

Primary mouse microglia were exposed to 20 or 40 nM catalytic MMP10 (cMMP10; Enzo), PBS (VEH), 25 μM GM6001 (pan-MMP inhibitor; Tocris) + cMMP10, or the positive control LPS (100 ng/mL; Invivogen) in MGM containing 1% FBS (Thermo Fisher) for 24 h in a volume 0.5 mL MGM (24-well). For experiments with treatment of ASC-specks, cells were untreated or stimulated with macrophage derived ASC speck (43.6 μg/mL) in MGM containing 5% FBS in 0.2 mL MGM (24-well) for 12 h as ASC-specks have been investigated previously at this dose and time-point in microglia (Franklin et al., 2014; Friker et al., 2020). The concentration of the ASC specks was estimated with a Pierce BCA Protein Assay Kit (Thermo Fisher) that was read with the Biotek Synergy H1 microplate reader.

### Enzyme-linked immunosorbent assays

The protein levels of mouse TNFα, total MMP-3, and IL-1β were determined with enzyme-linked immunosorbent assays (ELISAs) according to the manufacturer’s instructions (R&D Systems). Briefly, 50 μL of undiluted cell culture media was placed into each well, which were pre-coated with capture antibody. After a 2 h incubation, washes were performed followed by the addition of an enzyme-linked polyclonal antibody specific for the mouse antigen. After a 2 h incubation, washes were conducted again. Wells were incubated with substrate solution for 30 min and immediately read after the addition of stop solution with the Biotek Synergy H1 microplate reader.

### Mesoscale discovery

Levels of numerous pro-inflammatory cytokines were quantified with the V-Plex Mouse Proinflammatory Panel 1 Mouse Kit according to instructions provided by the manufacturer with minor modifications (MesoScale Discovery). Briefly, 50 μL of undiluted cell culture media was placed into each well, which were pre-coated with capture antibody. After a 2 h incubation, two washes were performed followed by the addition of a detection antibody for 2 h. Washes were repeated, and plates were subsequently developed and read using the Mesoscale Discovery MESO QuickPlex SQ 120 system.

MMP10 protein levels were measured in murine microglia with the human MMP10 R-plex MesoScale Discovery kit according to instructions provided by the manufacturer (MesoScale Discovery) since tools to detect MMP10 are limited and MMPs are well conserved in mammals (Zhang et al., 2006; Page-McCaw et al., 2007; Page-McCaw, 2008). Minor modifications were made to optimize the kit for cell culture. Briefly, 25 μL of undiluted cell culture media was placed into each well, which were pre-coated with capture antibody. After a 1 h incubation, two washes were performed followed by the addition of a detection antibody for 1 h. Washes were repeated, and plates were subsequently developed and read using the Mesoscale Discovery MESO QuickPlex SQ 120 system. To determine that MMP10 levels were not elevated due to the cMMP10 that was added externally to the media, a sample of alone was spiked with 40 nM cMMP10. The sample was subsequently added to the MSD plate and measured in the exact same way as the conditioned media (without any cells). The amount of exogenous cMMP10 that was detected by the kit was considered background from treatment and negligible compared to the MMP10 measured in the cMMP10 treated cells.

All MSD data was analyzed with Discovery Workbench 4.0. All measurements were performed in three independent experiments. Each experiment had three technical replicates per treatment.

### Iba1 immunocytochemistry

Cells were subjected to a 5 min wash with 1× phosphate buffered saline (PBS) and fixed for 20 min with PBS containing 4% (w/v) paraformaldehyde and 4% (w/v) sucrose, pH 7.4 at room temperature. Incubation for 5 min with PBS containing 0.1% (v/v) Triton X-100 was done to permeabilize cells. Microglia were subsequently blocked for 1 h with PBS containing 10% (v/v) goat serum and incubated overnight at 4°C with rabbit Iba-1 (1:750; Wako) in blocking buffer containing 10% goat serum. After incubation with Alexa Fluor 546 goat IgG secondary antibody (1:1000) in PBS containing 0.1% (v/v) triton X-100 and 1% goat serum, cells were counterstained with 4′,6-diamidino-2-phenylindole (DAPI; 13.0 ng/μL) in PBS, followed by two 5 min PBS washes (Daniele et al., 2015; Sánchez and Maguire-Zeiss, 2020). Coverslips were subsequently mounted with Hydromount (Electron Microscopy Services) in preparation for imaging with the Nikon Eclipse Ti-S inverted fluorescent microscope.

### Quantification of microglia morphology

Iba1 positive microglia were imaged and analyzed as previously established (Sánchez and Maguire-Zeiss, 2020). Five unique regions from each coverslip were captured at random by a blinded observer. To ensure that all areas of the coverslip were accurately shown, each coverslip was imaged in a snake-like pattern. To quantify cell morphology, Image J software (National Institute of Health) was used. Briefly, image type was transformed to 8-bit, followed by the adjustment of each image to a uniform threshold. Note that threshold was optimized so that the entire area of the whole cell was included. Each cell was then manually selected with the wand tool to collect its measurements. These Image J measurements included whole cell body area, Feret ratio (minimum Feret/maximum Feret), roundness (4 × area/π× major axis^2^ or inverse of aspect ratio), and circularity (4π × area/perimeter ^2^). Note that according to ImageJ, maximum Feret is the greatest distance between any two points of a selected object while minimum Feret is the shortest distance. Cells were not included in the analysis if they were Iba1 negative, lacked a nucleus, or had more than one nucleus. A minimum of 3–4 technical replicates (wells) per treatment were used in one independent experiment to evaluate microglia morphology. Values from individual cells is reported here instead since cells are not homogenous in primary mouse cultures. Values from individual cells are represented on the graphs and were used to conduct the statistical tests as described below.

### Quantification of microglia phenotype

A blinded observer manually classified microglia phenotype based on the number of processes on each Iba1 positive cell using the images obtained to evaluate morphology. The cells were organized into three categories; rod-like (2–3 processes small cell body), ameboid (large cell body no processes), and complex (4 or more processes) since these categories are based on previously established literature (Ayoub and Salm, 2003; Fernández-Arjona et al., 2017; Martini et al., 2020). This data was used to calculate the number of cells that were not rod-like in each frame or image.

### Statistical analysis

All experiments were performed a minimum of three times independently unless otherwise noted. Each experiment contained at least three technical replicates for each treatment group. GraphPad Prism, Version 8 was used to analyze all data. Either a Student’s *t*-test or one-way analysis of variance (ANOVA) was used to analyze data for all experiments excluding the LPS group, which was used as a positive control. The appropriate statistical test was selected based on the number of groups of interest analyzed. For the comparison of two groups a Student’s *t*-test was utilized while a one-way ANOVA was employed to compare three experimental groups. Since cells in primary murine cultures lack uniformity, data points shown represent individual cells for morphology as established in previous literature. More specifically, the average value for each well was not used due to this lack of homogeneity (Allen et al., 2016; Hoenen et al., 2016; De Biase et al., 2017; Winland et al., 2017; Abdolhoseini et al., 2019; Svoboda et al., 2019). For every analysis, significance threshold was set at *P* ≤ 0.05. Data are represented as mean ± SEMS.

## Results

### Exposure to the inflammasome adaptor protein ASC can induce morphological changes in microglia

**FIGURE 1:**
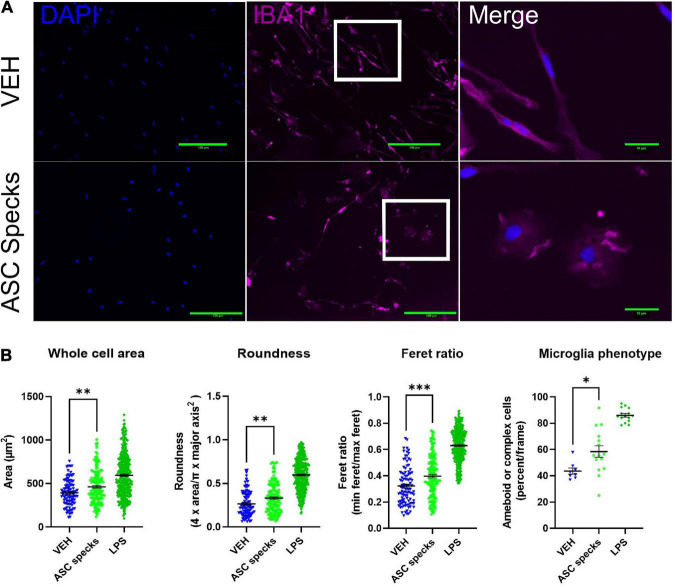
Aggregated ASC specks alter microglia shape. Iba1 immunocytochemistry was conducted to quantify cell morphology. **(A)** Microglia were stimulated with ASC specks (43.6 μg/mL) or vehicle (VEH) for 12 h. Microglia structure was visualized with Iba1 (magenta) while the nucleus was stained with DAPI (blue). Microglia that are enlarged are indicated by the white box and shown in the far-right column of each row. **(B)** Consistent with a modification in shape, ASC speck treatment resulted in a morphological shift in microglia. The experiment was conducted one time with three technical replicates (wells) and the values reported are the measurement from each individual cell; VEH *n* = 103 cells, ASC speck *n* = 147 cells, and LPS = 360 cells. The percentage of proinflammatory microglia per frame is also altered by treatment; VEH *n* = 8 images, ASC speck *n* = 15 images, and LPS = 15 images). Values are reported as mean ± SEM; Unpaired Student’s *t*-test compared to VEH; *P ≤ 0.05, ^**^P ≤ 0.01, and ^***^P ≤ 0.001.

Since it is well established that microglia respond to their environment by adopting an ameboid shape in the presence of inflammatory stimuli such as LPS, we wanted to evaluate the impact of enriched ASC aggregates (ASC-specks) on microglia morphology (Torres-Platas et al., 2014; De Biase et al., 2017; Winland et al., 2017). To that end we examined the consequences of ASC-specks treatment on microglial shape 12 h post-treatment. Insoluble ASC-specks cause microglia display a more complex or ameboid shape compared to VEH (Figure 1A). When quantified, ASC-specks display a significantly elevated cell body area, roundness, and Feret ratio. This finding was corroborated since ASC-speck stimulated microglia also display a higher percentage of ameboid or complex cells (Figure 1B).

### Microglial exposure to ASC-specks stimulates matrix-metalloproteinases release

**FIGURE 2:**
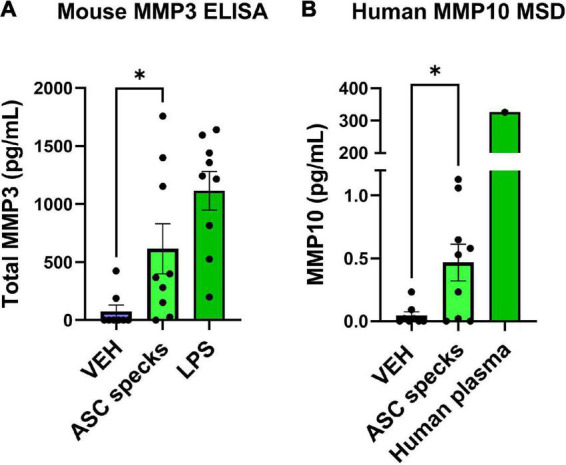
Purified ASC speck aggregates stimulate the release of MMPs. ELISA and MSD were performed on supernatants derived from microglia after ASC speck or VEH stimulation. **(A)** Microglia treated with purified ASC specks (43.6 μg/mL) for 12 h increased the release of MMP3 (*n* = 9 wells/treatment). LPS treated microglia are shown and served as a positive control. **(B)** MMP10 is also released by these cells as determined by MSD (*n* = 9–8 wells/treatment). One human plasma sample served as a positive control. The values shown are derived from three independent experiments in which there were three technical replicates, or wells, per treatment. Each independent experiment is shown in the graph and was statistically analyzed together. Values are reported as mean ± SEM; Unpaired Student’s *t*-test; *P ≤ 0.05.

Since ASC-specks are elevated in patients with AD and can alter microglial morphology, further studies were warranted. Given that the IL-1β regulates MMP release (Lew et al., 2018), the impact of ASC-specks was evaluated 12 h post-exposure in microglia (Franklin et al., 2014). MMP3 and MMP10 release by primary microglia was measured with ELISA and MSD respectively (Figure 2). Stimulation with ASC-specks (43.6 μg/mL) resulted in the statistically significant release of MMP3 compared to VEH (Figure 2A). LPS served as a positive control for MMP3 release. MMP10 was also elevated upon stimulation of microglia with purified ASC-specks and was significantly higher compared to VEH treated cells based on MSD analyses (Figure 2B). One human plasma sample served as a positive control for the MMP10 measured with MSD. Overall, the elevation in MMPs demonstrated that ASC-specks treatment can stimulate MMP release and that microglia are a source of MMP10.

### Microglial stimulation with cMMP10 results in morphological alterations that can be reversed with a pan-matrix-metalloproteinases inhibitor

**FIGURE 3:**
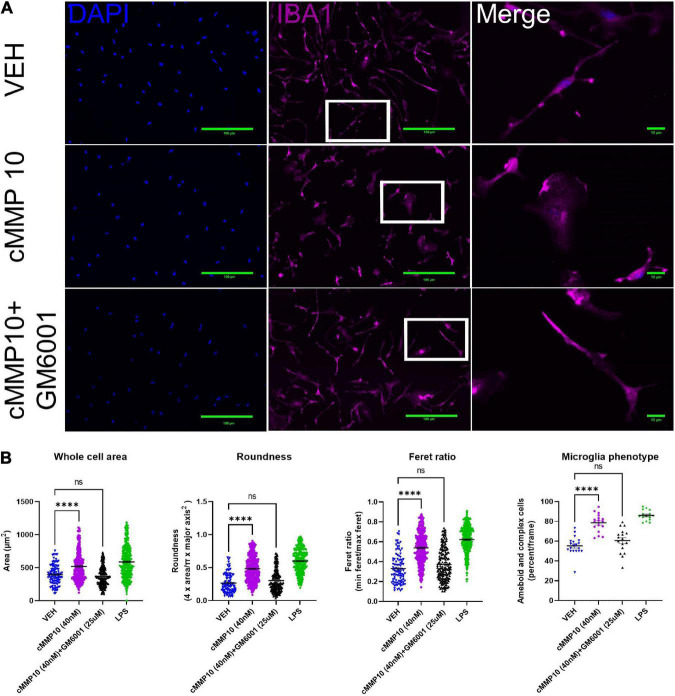
cMMP10 induced morphology changes are reversed in microglia with the pan-MMP inhibitor GM6001. **(A)** Iba1 immunocytochemistry (magenta) was conducted on microglia to analyze cell morphology after exposure to 40 nM cMMP10 or vehicle (VEH) for 24 h. Microglia that are enlarged are indicated by the white box and are shown in the far-right column of each row. **(B)** Consistent with a visual change, cMMP10 exposure led to a change in morphological parameters such as whole cell area, roundness, and Feret ratio compared to vehicle treatment; VEH *n* = 103 cells, cMMP10 *n* = 340 cells, cMMP10 + GM6001 = 184 cells, and LPS = 360 cells. cMMP10 exposure also increased the percentage of proinflammatory microglia (ameboid or complex); VEH *n* = 19 images, cMMP10 *n* = 18 images, cMMP10 + GM6001 = 17 images, and LPS *n* = 15 images. Notably these changes are reversed by the pan-MMP10 inhibitor GM6001 (no significance compared to VEH). LPS was used as a positive control. Through the experiment was conducted with three technical replicates per treatment, values reported are the measurement from individual cells. When the average of each well is used to perform statistical analysis instead, the trend observed still holds. Values are reported as mean ± SEM; One-way ANOVA compared to VEH; Dunnett’s *post-hoc* test; ^****^P ≤ 0.0001.

Since MMP10 is elevated in the central nervous system of dementia patients (Erhardt et al., 2021), the next step was to evaluate the consequences of ASC-specks mediated release of cMMP10 on microglia. To that end, the impact of cMMP10 (40 nM) was assessed by evaluating the shape of murine cortical microglia 24 h post-stimulation (Figure 3). To determine the impact of MMP-inhibition, microglia were also stimulated for 24 h with recombinant catalytic MMP10 (cMMP10; 40 nM) alone or catalytic MMP10 with the pan-MMP inhibitor GM6001. Iba1 immunocytochemistry and ImageJ analyses was used to determine microglia shape as previously described (Sánchez and Maguire-Zeiss, 2020; Figure 3A). cMMP10 induced a statistically significant change in all morphometric measurements, and those alterations were reversed after GM6001 stimulation (Figure 3B). LPS served as a positive control. Whole cell body area, roundness and Feret ratio were higher in vehicle-treated cells compared to cMMP10-stimulated microglia indicative of a rounder appearance (Figure 3B). Lastly, cMMP10 increases the percentage of ameboid or complex microglial compared to VEH. Taken together, changes in these morphological indicate that cells no longer resemble a rod and now have an ameboid shape. These morphological changes were reversed with the pan-MMP inhibitor GM6001, as whole cell area, roundness, and Feret ratio of cells treated with cMMP10 + GM6001 were not significantly different from VEH. GM6001 is not significantly different from VEH (not shown). Overall, these morphological changes indicate a shift in cMMP10-stimulated microglia to a phagocytic shape which likely indicate functional changes in the microglia.

### Microglial exposure to cMMP10 stimulates the release of proinflammatory cytokines

**FIGURE 4:**
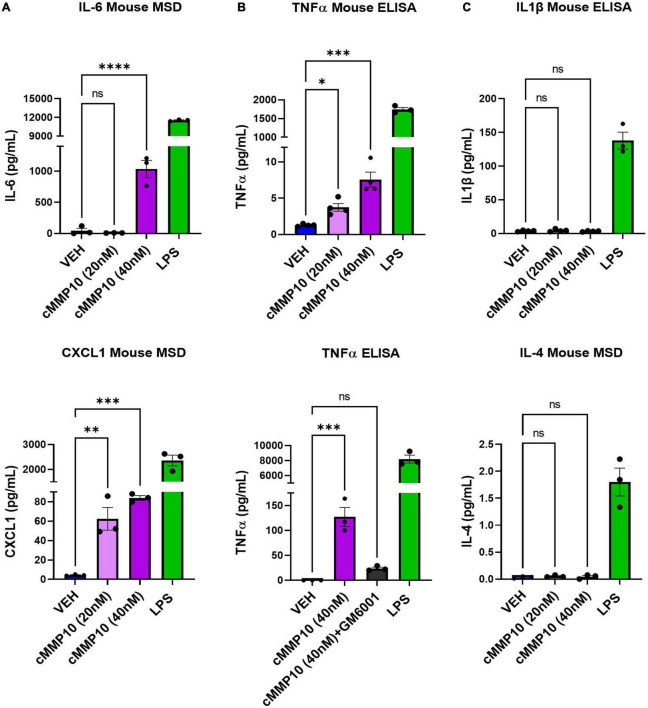
The release of classical proinflammatory cytokines is increased in cMMP10-stimulated microglia. Microglia were exposed to cMMP10 (20 or 40 nM) or vehicle (VEH) for 24 h. **(A)** Cells stimulated with 40 nM cMMP10, but not 20 nM cMMP10, significantly increase IL-6. These cells also release CXCL1 release in a dose responsive manner to cMMP10. **(B)** cMMP10-exposed microglia demonstrate higher levels of TNFα in a dose responsive manner compared to VEH treated cells (ELISA; *n* = 4 wells/treatment). **(B)** The increase of TNFα following cMMP10 exposure was reversed when cMMP10 was incubated with the pan-MMP10 inhibitor GM6001 (ELISA; *n* = 3 wells/treatment). **(C)** cMMP10 is not sufficient to stimulate the release of IL-1β or IL-4. Values reported are representative of one experiment performed with three technical replicates, or wells, per treatment. The experiment was conducted independently two additional times. Values are reported as mean ± SEM; One-way ANOVA compared to VEH; Dunnett’s *post-hoc* test; ^****^P ≤ 0.0001, ^***^P ≤ 0.001, ^**^P ≤ 0.01, and *P ≤ 0.05.

After establishing that microglial morphology is altered by cMMP10 treatment, we sought to better comprehend the functional implications of cMMP10 on cytokine release. To that end, we evaluated the release of proinflammatory proteins twenty-four hours after exposure to cMMP10. A statistically significant increase in the levels of the proinflammatory cytokine IL-6 and chemokine CXCL1 was detected in microglia treated with 40 nM of MMP10, with CXCL1 being released in a dose responsive manner (Figure 4A). TNFα was also observed in microglia upon stimulation with both 20 and 40 nM cMMP10 compared to vehicle-control cells (Figure 4B). Moreover, the release of TNFα was reversed when cMMP10 was incubated with GM6001, as TNFα levels in this group were not significantly compared to VEH (Figure 4B). Note that TNFα release in cells treated with GM6001 alone was undetectable (not shown). In contrast, unlike LPS-induced IL-1β and IL-4 release, stimulation of microglia with either dose of cMMP10 did not result in the secretion of IL-1β or IL-4 secretion (Figure 4C). LPS served as a positive control for the release of all proinflammatory factors shown.

### cMMP10 mediated changes on microglia lead to the continued release of MMP10, but not other stromelysins

**FIGURE 5:**
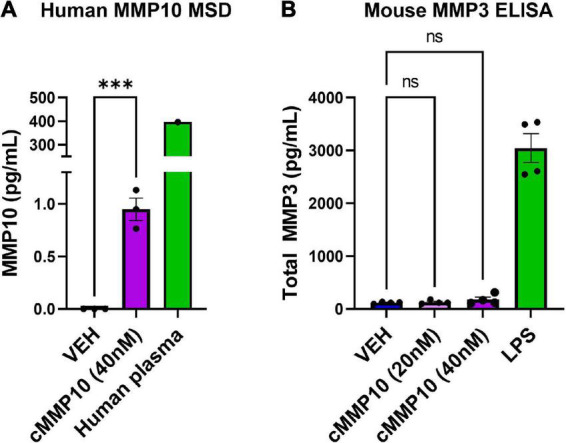
Microglia release more MMP10 in response to cMMP10 treatment. ELISAs and MSD were conducted with supernatants collected after exposure to cMMP10 or VEH. **(A)** Microglia treated with cMMP10 (40 nM) for 24 h increased the release of MMP10 as measured by the MMP10 MSD R-plex (*n* = 3–4 wells/treatment). One human plasma served as a positive control. **(B)** Microglial MMP3 release did not occur in response to MMP10 at any dose (*n* = 4 wells/treatment; no significance). LPS served as a positive control. Values reported are representative of one experiment that had a minimum of three biological replicates, or wells, per treatment. This experiment was performed independently at least two additional times. Values are reported as mean ± SEM; Unpaired Student’s *t*-test (MSD) or one-way ANOVA compared to VEH (ELISA) Dunnett’s *post-hoc* test; ^***^P ≤ 0.001.

Since MMP10 activates the release of other MMPs in certain cell types, we wanted to determine if MMP10 stimulates microglial release of stromelysins. More specifically, we wanted to evaluate if cMMP10 could lead to the release of MMP3 or even the autocrine release of MMP10 itself in microglia (Knäuper et al., 1997; Lausch et al., 2009). To that end, first we noted that autocrine-effect and release of MMP10, with an overall increase in the release of MMP10 compared to VEH (Figure 5A). One human plasma sample served as a positive control for MMP10 detection with MSD. In contrast, MMP3 production was not affected when quantified 24 h post-cMMP10 treatment compared to vehicle-treated microglia (Figure 5A). These microglia were capable of releasing MMP3, as LPS-stimulation induced MMP3 release (Figure 5B).

## Discussion

**FIGURE 6:**
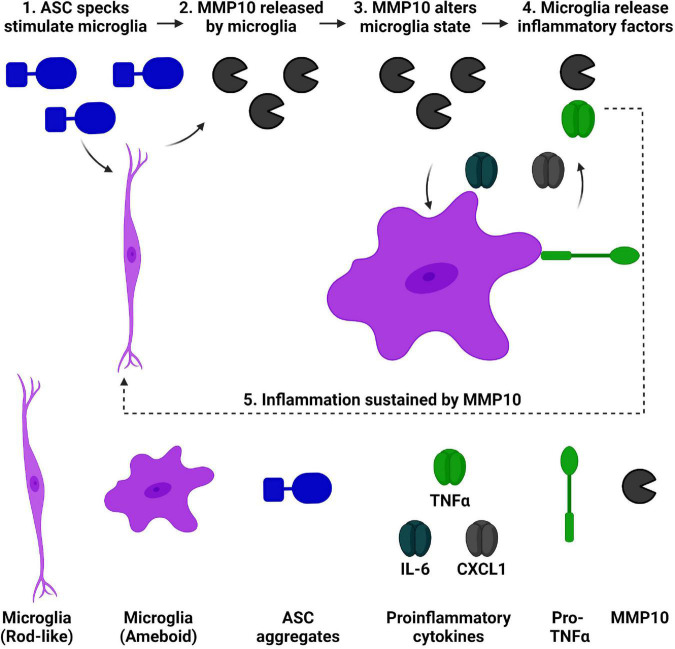
ASC speck-mediated of release of MMP10 may perpetuate inflammation of microglia. We have established that ASC-speck aggregates can stimulate the release of MMP10 in primary microglia. As a result of exposure to MMP10, adjacent microglia will take on a pro-inflammatory state. This phenotype includes altered morphology and the release of inflammatory factors TNFα, IL-6, and CXCL1. In addition to classical proinflammatory cytokines, even more MMP10 is released so that it can act on adjacent microglia to continue the cycle of inflammation since IL-4 is not present. Taken together, these findings suggest that MMP10 perpetuates a chronic inflammatory state. Image created with BioRender.

Matrix-metalloproteinases are elevated in dementia, and downstream consequences of their activity are relevant to consider. Recently, we found that both MMP10 and the assembled inflammasome complex ASC-specks are elevated in the CSF of dementia patients (Erhardt et al., 2021; Jiang et al., 2021). Microglial cell cultures were utilized to characterize the inflammatory consequences of ASC-speck mediated release of MMP10. Importantly, a novel role of the inflammasome-adaptor protein, ASC, and its assembled form (ASC-specks or pyroptosomes), was identified. Here, we show for the first time that assembled ASC-specks can increase the release of MMPs in primary microglia. We found an increase in MMP3 and MMP10 levels of microglia after ASC-specks exposure. We also ascertained the consequences of ASC/inflammasome-mediated MMP10 release on microglia. More specifically, we found that MMP10 stimulation altered microglial phenotype, suggesting that MMP10 sustains inflammation (Figure 6). Its ability to perpetuate continual inflammation is critical to the chronic activation state of microglia observed in disease since it alters their ability to harness autoimmunity (McGeer et al., 1988; Schwartz et al., 2003; McGeer and McGeer, 2008; Perry et al., 2010; Perry and Teeling, 2013).

Our findings indicate that ASC-speck exposure induces morphological changes and stimulates the release of both MMP10 and MMP3 in microglia. The ability of ASC-specks to influence microglia phenotype corroborates previous work. For example, ASC-specks can be released extracellularly and propagate from cell to cell like a prion in the presence of Danger Associated Molecular Patterns (DAMPs) such as amyloid-β and phosphorylated tau (Franklin et al., 2014; Venegas et al., 2017; Jiang et al., 2021). As a consequence, ASC-specks are taken up by adjacent immune cells and trigger a phagocytic phenotype that culminates in the release of IL-1β (Baroja-Mazo et al., 2014; Franklin et al., 2014; Venegas et al., 2017). Taken together, both our findings and previous work suggests that ASC-specks are capable of inducing morphofunctional changes in microglia. Previous literature has also linked the inflammasome to MMP release. IL-1β transcriptionally regulates MMPs, for example MMP13 and MMP9, in multiple cell types (Cheng et al., 2010; Hashimoto et al., 2013; Zhang et al., 2018; Sánchez and Maguire-Zeiss, 2020). IL-1β leads to the release of MMP-1,9,13 in chondrocytes. Furthermore, MMP1 is upregulated by fibroblasts in response to IL-1β.

We also establish that MMP10 stimulation of microglia results in morphological and functional changes through a classical inflammatory pathways regulated by NF-κB such as IL-6, CXCL1, and TNFα (Libermann and Baltimore, 1990; Brasier, 2010; Burke et al., 2014; Daniele et al., 2015). This finding supports previous work that indicate MMPs can stimulate phenotypic changes in microglia. For example, MMP13-stimulation of primary mouse microglia leads to altered shape and the release of TNFα (Sánchez and Maguire-Zeiss, 2020). More relevant to this work, MMP3 is a second example more closely related to MMP10 since it is another stromelysin that is capable of impacting microglia (Connolly et al., 2016; Hu et al., 2016; Brelstaff et al., 2021). Due to the previously established literature related to MMP3, MMP10 was investigated further in our work instead. MMP3 exposure stimulates IL-6 release in AC128 microglial cells and TNFα in BV2 cells (Kim et al., 2005; Connolly et al., 2016). More recently, MMP3 expression has been shown to be a marker of senescent microglia (Brelstaff et al., 2021).

There were other inflammatory factors notably absent after MMP10 stimulation. Often, it has been reported that MMPs can stimulate the release of other MMPs in immune cells (Knäuper et al., 1997; Lausch et al., 2009; Sánchez and Maguire-Zeiss, 2020). For example, in a similar study conducted, microglia released MMP9 in response to MMP13 stimulation (Sánchez and Maguire-Zeiss, 2020). Here we report that MMP10 does not have the capability to release MMP3 in microglia. More specifically, MMP3 was not released by cells though MMP10 was continually released. This finding supports the idea that MMP10 and MMP3 are functionally redundant since they are both stromelysins. Perhaps functional redundancy is why only one stromelysin was released upon MMP stimulation. Furthermore, while microglial activation has been previously viewed to be polarized that view is no longer held. In fact, microglia transcriptomics has established how they can express both proinflammatory and anti-inflammatory molecules at the same time (Chen and Trapp, 2016; De Biase et al., 2017; Li et al., 2021), which is a topic for further investigation given the context of current findings.

IL-1β and IL-4 were also not released by microglia upon cMMP10 stimulation. This finding is consistent with previous work related to MMP-induced inflammation of microglia. MMPs do not necessarily stimulate IL-1β release in microglia, as previously demonstrated with MMP13 (Sánchez and Maguire-Zeiss, 2020). Our finding further supports the possibility that MMP-mediated inflammation is inflammasome independent. However, future investigations can better determine that hypothesis. Furthermore, the absence of IL-4 indicates that the microglia are unable to stimulate a repair response to counteract cMMP10 mediated inflammation. In fact, it shifts microglia to a neuroprotective phenotype by influencing functions such as phagocytosis (Yi et al., 2020; Zhang et al., 2021).

Though inflammatory biomarkers have recently emerged as possible contributors to central nervous system damage, many studies have yet to understand the molecular relationship between proteins elevated in patient samples (Lorenzl et al., 2002, 2003; Annese et al., 2015; Connolly et al., 2016; Sánchez and Maguire-Zeiss, 2020; Erhardt et al., 2021). For example, both inflammasome adaptor proteins and MMPs are elevated in AD (Lorenzl et al., 2002, 2003; Antonell et al., 2020; Erhardt et al., 2021). Overall, the results presented here demonstrate that dementia outcomes may be improved through the pharmaceutical targeting of both MMPs and inflammasome related proteins (Caplan and Maguire-Zeiss, 2018). Though the development of MMP inhibitors has well documented obstacles, it is interesting that we were successful at inhibiting the detrimental effects of this proinflammatory activity with the drug GM6001 (Figures 4, 5). In fact, microglia no longer released TNFα or transformed their morphological phenotype. Both clinically relevant MMP and inflammasome inhibitors warrant further investigation (Allen et al., 2016; Bozzelli et al., 2019; Giriwono et al., 2019). Though this work has described the autocrine effects of MMP10 on microglia, MMP10 target(s) in microglia is yet to be identified. In fact, the TNFα is known to be one of the potential targets of MMP10 cleavage. This is possible given that MMPs are capable of cleaving TNF and that MMP10 stimulation led to the release of TNFα (Könnecke and Bechmann, 2013). One other possible MMP10 target we may study in the future is TREM2 (Guerreiro et al., 2013; Jonsson et al., 2013; Sims et al., 2017; Ulland and Colonna, 2018). *TREM2* variants increase the risk of late onset AD by 2–4-fold, and TREM2 is essential to the metabolic shift related to mTOR signaling in inflammatory microglia (Guerreiro et al., 2013; Sims et al., 2017; Ulland and Colonna, 2018). More pertinent to this study, TREM2 protein levels correlate with MMP10 levels in patients with dementia (Whelan et al., 2019). After identification of a target, potential pathways downstream of the target would also be interesting to examine given the molecular mechanisms linked to altered MMP activity in immune cells. More specifically, tau-mediated release of MMP3 results in decreased phagocytic activity in microglia. This occurs through, both the NF-κB and senescence pathways of microglia, providing evidence that MMP10 should be evaluated in a similar context in the future given the findings of this study (Brelstaff et al., 2021). More specifically, pathways of interest would include those related to TLR signaling (MyD88, NF-κB, etc.) phagocytosis (CD68, TREM2, CX3CR1, etc.), and senescence (P19ARF). Given the limitations of *in vitro* models used in this paper, fully understanding the contribution of specific MMP10 to disease in humans necessitates the use of specific inhibitors and animal models in future studies. For example, MMP10 knockout mice will be used to further corroborate the MMP inhibitor studies. Due to the well-established relationship between IL-1β and MMPs, it is also important to consider that MMP-mediated damage could be prevented by inhibiting inflammasome adaptor proteins. Based on this, future studies will investigate the pathways related to ASC-mediated MMP release. By conducting the proposed investigations, pharmaceutical interventions may be developed in AD.

## Data availability statement

The raw data supporting the conclusions of this article will be made available by the authors, without undue reservation.

## Ethics statement

The animal study was reviewed and approved by University of New Mexico IACUC.

## Author contributions

KS wrote the initial draft and performed all experiments. KB provided feedback on scientific interpretation and edited drafts. GR and KS planned and interpreted experiments. All authors contributed to the article and approved the submitted version.
